# Endoscopic nasobiliary drainage for obstructive jaundice using either a 5 Fr or 7 Fr catheter: a prospective, randomized trial

**DOI:** 10.1186/1471-230X-14-161

**Published:** 2014-09-18

**Authors:** Toshio Fujisawa, Koichi Kagawa, Shunsuke Watanabe, Kantaro Hisatomi, Kensuke Kubota, Hajime Sato, Atsushi Nakajima, Nobuyuki Matsuhashi

**Affiliations:** Department of Gastroenterology, NTT Medical Centre Tokyo, 5-9-22 Higashi-Gotanda, Shinagawa, Tokyo, 141-8625 Japan; Gastroenterology Division, Yokohama City University School of Medicine, Yokohama, Japan; Department of Health Policy and Technology Assessment, National Institute of Public Health, Saitama, Japan

**Keywords:** Nasobiliary catheter, Obstructive jaundice, Endoscopic biliary drainage, Questionnaire survey, Catheter size

## Abstract

**Background:**

The influence of size on the effectiveness of nasobiliary catheters has not yet been studied. We compared biliary drainage effectiveness and procedure-related discomfort and adverse events in 5 French (Fr) and 7 Fr nasobiliary catheters.

**Methods:**

We prospectively studied 100 patients undergoing endoscopic biliary drainage for obstructive jaundice, who were randomly allocated to a 5 Fr or 7 Fr nasobiliary catheter group. As the primary endpoint, the effectiveness was evaluated by the serum total bilirubin decreasing rate and the success rate of jaundice relief. As the secondary endpoint, the degree of discomfort was investigated using a questionnaire survey after catheter removal.

**Results:**

The bilirubin decrease rate was significantly higher in the 7 Fr catheter group than in the 5 Fr group (53.0 ± 21.4% vs 40.5 ± 29.9%, respectively; *P* = 0.019). The success rate of jaundice relief tended to be higher in the 7 Fr catheter group, although the difference was not statistically significant (98% vs 88%, respectively; *P* = 0.056). The questionnaire survey demonstrated that total discomfort was significantly greater in the 7 Fr group (3.9 ± 1.5 vs 3.2 ± 1.4, respectively; *P* = 0.018). Larger-diameter catheters tended to increase difficulty in eating, although the difference between the groups was not statistically significant.

**Conclusions:**

7 Fr nasobiliary catheters are recommended for patients requiring rapid and reliable relief of obstructive jaundice. However, because they can cause greater discomfort, 5 Fr nasobiliary catheters are preferred in other settings.

**Trial registration:**

On July 1, 2012; UMIN000008288 (Japan Primary Registries Network).

**Electronic supplementary material:**

The online version of this article (doi:10.1186/1471-230X-14-161) contains supplementary material, which is available to authorized users.

## Background

There are two methods of endoscopic biliary drainage for obstructive jaundice and acute cholangitis. One is a nasobiliary catheter and the other is a biliary stent [1]. Both strategies appear to be equally effective for biliary drainage [2–4]. However, each method has its advantages and disadvantages. Nasobiliary catheters can be inserted easily, quickly, and safely. Moreover, the condition of bile can be monitored, and cholangiography can be performed using these catheters. Bile cytology via a nasobiliary catheter could also improve diagnostic sensitivity for malignancy [5]. In preoperative drainage, cholangitis due to tube occlusion happens less frequently with nasobiliary catheters than with biliary stents [6]. Removal of nasobiliary catheters is also easy. However, nasobiliary catheters may be dislodged or removed by patients with delirium or dementia. Kinking of these catheters may also occur, which prevents effective biliary drainage. Furthermore, nasobiliary catheters produce discomfort and are cosmetically unappealing, as they exit at the nostril and wind around the face. Patients with a nasobiliary catheter must bring a collecting bag with them at all times [7]. On the basis of these characteristics, nasobiliary catheters are generally preferred in Eastern countries and biliary stents are usually preferred in Western countries.

Sharma et al. compared the effectiveness of biliary drainage using 7 Fr and 10 Fr biliary stents in patients with acute cholangitis and reported that both sizes of stents were equally effective [8]. Ishigaki et al. compared complications between 4 Fr and 6 Fr nasobiliary catheter groups and reported less frequent post-endoscopic retrograde cholangiopancreatography (ERCP) pancreatitis in the 4 Fr catheter group [9]. However, the influence of catheter gauge on the effectiveness of nasobiliary catheters has not yet been studied. Therefore, we conducted this prospective, randomized, controlled trial to compare 5 Fr and 7 Fr nasobiliary catheters. We investigated the effectiveness of bile duct drainage using these catheters as the primary endpoint, and discomfort or other problems related to the nasobiliary catheters as the secondary endpoint.

## Methods

### Study design

This study was a prospective, randomized, controlled single-centre trial conducted by multiple endoscopists. Given our experience that the overall success rate of drainage treatment was around 94%, and that a catheter with a larger diameter was expected to be more efficacious in drainage treatment, the sample size was calculated so that the study could show that the average treatment success rate with 7 Fr catheter is larger than that with 5 Fr catheter more than 40% at alpha = 0.05 with power = 0.80.

Enrolled patients were assigned to two groups: 5 Fr nasobiliary catheter or 7 Fr nasobiliary catheter. Fifty patients were allocated to each of the two groups, and a total of 100 patients were included in the study. Randomization of patients was performed according to a random number table, which was established before the study began. The primary outcome was the effectiveness of biliary drainage. The secondary outcome was the presence of catheter-related discomfort or other problems. Single-blind method was applied. The patients were blinded to the assigned group. The organizer (T.F.) informed the operators of the assignment, but did not take part in the decision of the therapeutic strategy. Two dedicated ERCP trainees (S.W. and K.Kagawa) performed the ERCPs, and at least one senior endoscopist, whose career spanned more than 10 years, directly supervised all procedures (K.H. and K.Kubota). These operators were not informed about the group assignment until immediately before the nasobiliary catheter was inserted. The study protocol was approved by the ethics committee of NTT Medical Centre Tokyo and was in accordance with the guidelines of the Declaration of Helsinki for biomedical research involving human subjects. This trial was registered on July 1, 2012 in the Japan Primary Registries Network (registration number: UMIN000008288), which is a member of World Health Organization Registry Network. Recruitment of participants began in July 2012 and was completed in October 2013.

### Eligibility criteria

We recruited patients with jaundice and an elevated total serum bilirubin (≥2 mg/dL) caused by obstruction of the common bile duct. The exclusion criteria were as follows: age younger than 20 years, separated bile duct due to hilar obstruction, bile duct obstructed at multiple sites, liver failure, contrast medium allergy, previous Billroth II gastrectomy or Roux-en-Y reconstruction, pregnancy, or refusal to provide informed consent.

### Study protocol

ERCP was performed using a JF-260 V or TJF-260 V duodenoscope (Olympus Medical Systems Corp, Tokyo, Japan). After selective deep cannulation into the common bile duct, bile was aspirated to confirm the proper position of the cannula. Injection of contrast medium was avoided as far as possible to prevent cholangiovenous reflux. Endoscopic papillary balloon dilation (EPBD) or endoscopic sphincterotomy (EST) was performed for all patients in the primary session except who were taking anticoagulant/antiplatelet drug or showing hemorrhagic diathesis. EPBD was performed for removing stones and placing a plastic stent into the bile duct. EST was selected for placing self-expandable metallic stent over the papilla. Either a 5 Fr or a 7 Fr nasobiliary catheter was placed into the left or right hepatic duct using a 0.035-inch guide wire (Hydrajagwire 5605; Boston Scientific Corp, Natick, MA). Both sizes of the catheters were 255 cm long, tapered, pig-tail tipped, with 9 side holes from the tip and with alpha loop in the duodenal portion (Olympus Medical Systems Corp, Tokyo, Japan). The causes of obstructive jaundice were left untreated in the first session and were treated in the second session after alleviation of the cholangitis or enough decompression of the bile duct. Prophylactic pancreatic stents were inserted in patients that underwent a papillary manipulation. 5 Fr straight polyethylene stents, 3 cm in length, unflanged on the pancreatic duct side, and with two flanges on the duodenal side (GPDS-5-3; Cook Endoscopy Inc., Winston-Salem, NC) were used. Stent dislodgment was confirmed by abdominal radiography before discharge. Procedure-related adverse events and incidents were recorded according to the definitions and grading systems suggested by the 2010 workshop held by the American Society of Gastrointestinal Endoscopy workshop in 2010 [10]. Serum total bilirubin (TB), alkaline phosphatase (ALP), and gamma-glutamyl transpeptidase (γGTP) were measured at least 3 times: before the procedure (day 0), on day 1 after the procedure, and on day 4 after the procedure. The efficacy of bile duct drainage was evaluated by two indicators: the decreasing rate of each parameter (TB, ALP, and γGTP) and the success rate of jaundice relief. The decreasing rate of each parameter was calculated by the following formula: [(Day0 - Day4)/Day0] × 100, in which Day0 and Day4 represent the values of each parameter on day 0 and day 4, respectively. The success rate in jaundice relief was defined as the proportion of patients in which Day4 value was lower than Day0. After removal of the catheter, the patients completed a questionnaire survey about their experiences with the nasobiliary catheter therapy (Additional file 1: Figure S1). The survey asked the patients to use a visual analogue scale (VAS; 0, none; 10, maximum) to rate their overall total discomfort, as well as their degree of difficulty in eating. Subjective symptoms including nasal haemorrhage, sore throat, nausea, caught on a nearby object, or other complications related to the nasobiliary catheter were also investigated. The volume of bile drainage on days 1, 3, and 5 after the procedure was recorded, and the average volume per day (mL/day) was calculated. In patients with acute cholangitis, the periods to alleviation of fever after the nasobiliary catheter placement were investigated every 12 hours and were compared between the 5 Fr and 7 Fr catheter groups. The result is shown in a Kaplan-Meier method

### Statistical analysis

Quantitative data were expressed as the mean ± standard error. Student *t* tests were used to compare continuous variables between the two groups after normal distribution of the data was verified by Kolmogorov-Smirnov test. Chi-squared tests and two-tailed Fisher’s exact tests were used to analyse clinical variables between the two groups (the Fisher’s exact test was used when the numbers were small). Log-rank test was used to analyse the difference of the periods to alleviation of fever. A *P* value less than 0.05 was considered statistically significant. All statistical analyses were performed using PASW statistics version 20 (IBM Corporation, Armonk, NY).

## Results

**Figure 1:**
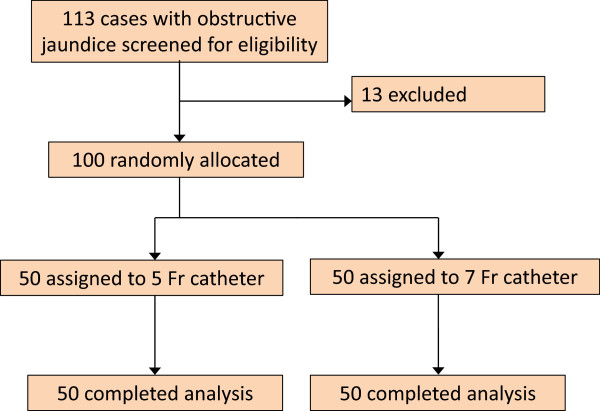
**Flow diagram of the trial.** After random allocation, all patients underwent endoscopic biliary drainage with either a 5 Fr nasobiliary catheter or 7 Fr nasobiliary catheter.

A total of 113 patients with obstructive jaundice were screened to determine if they were eligible to participate in the study. Thirteen patients were excluded because of the presence of exclusion criteria: separated bile duct due to hilar obstruction, 4 patients; bile duct with multiple sites of obstruction, 3 patients; previous Billroth II gastrectomy, 2 patients; previous Roux-en-Y reconstruction, 2 patients; and refused to provide informed consent, 2 patients. After random allocation, all patients underwent endoscopic biliary drainage with a 5 Fr or 7 Fr nasobiliary catheter, and their data were subsequently analysed (Figure 1).

Of the 100 patients enrolled in the study, 50 were randomized to the 5 Fr catheter group (25 men, 25 women; mean age, 75.1 ± 1.5 years) and 50 were randomized to the 7 Fr catheter group (31 men, 19 women; mean age, 71.3 ± 1.9 years). The primary cause of the obstructive jaundice was as follows: choledocholithiasis, 45 patients; cholangiocarcinoma, 25 patients; pancreatic cancer (including intraductal papillary mucinous carcinoma), 16 patients; hepatic lymph node metastasis, 8 patients; gallbladder cancer, 4 patients; autoimmune pancreatitis, 1 patient; and sclerosing cholangitis, 1 patient. Signs of acute cholangitis including fever, epigastric pain, or an elevated serum C-reactive protein, were observed in 22 patients. All patients recovered from acute cholangitis after bile duct drainage. For further treatment, the papilla of Vater was manipulated by EPBD in 66 cases and by EST in 12 cases. 18 cases taking an anticoagulant/antiplatelet drug and 4 cases showing haemorrhagic diathesis by disseminated intravascular coagulopathy did not undergo papillary manipulation. These 22 patients underwent a papillary manipulation in the second session of the treatment after heparinization or treatment for haemorrhagic diathesis. The manipulation methods were similar in the two groups. However, pancreatic stent insertion for prevention of post-ERCP pancreatitis was more common in the 7 Fr catheter group than in the 5 Fr catheter group (Table 1).

**Table 1:** **Clinical characteristics of patients undergoing biliary drainage**

	5 Fr catheter (n = 50)	7 Fr catheter (n = 50)	*P*
Gender (male/female)	25 (50)/25 (50)	31 (62)/19 (38)	0.227
Mean age ± standard error	75.1 ± 1.5	71.3 ± 1.9	0.129
Presence of cholangitis	13 (26)	9 (18)	0.334
Primary disease			0.957
Choledocholithiasis	23 (46)	22 (44)	
Cholangiocarcinoma	13 (26)	12 (24)	
Pancreatic cancer	8 (16)	8 (16)	
Hepatic lymph node metastasis	4 (8)	4 (8)	
Gallbladder cancer	1 (2)	3 (6)	
Others	1 (2)	1 (2)	
Management of papilla			0.448
EPBD	30 (60)	36 (72)	
EST	7 (14)	5 (10)	
none	13 (26)	9 (18)	
Prophylactic pancreatic stent	22 (44)	35 (70)	0.009

EPBD: endoscopic papillary balloon dilation, EST: endoscopic sphincterotomy. Data were shown as the number of patients (percentage).

A 5 Fr or 7 Fr catheter was placed into the left or right hepatic duct of each patient, and endoscopic biliary drainage was successful in all 100 patients. The serum TB levels were similar in the 5 Fr and 7 Fr catheter groups before the procedure (5.4 ± 0.5 mg/dL vs 5.8 ± 0.8 mg/dL, respectively), at day 1 after the procedure (4.6 ± 0.6 mg/dL vs 4.2 ± 0.6 mg/dL, respectively), and at day 4 after the procedure (3.4 ± 0.5 mg/dL vs 2.9 ± 0.5 mg/dL respectively) (Table 2). However, the bilirubin decrease rate was significantly higher in the 7 Fr catheter group than in the 5 Fr catheter group (53.0 ± 3.0% vs 40.5 ± 4.2%, respectively; *P* = 0.019). The bilirubin decrease rate was also investigated separately in each primary disease, and the bilirubin decrease rate was higher in the 7 Fr catheter group than in the 5 Fr catheter group for every primary disease (Additional file 2: Figure S2). The success rate of jaundice relief (TB level) tended to be higher in the 7 Fr catheter group, although the difference between the groups did not reach statistical significance (98% vs 88% for the 7 Fr and 5 Fr catheter groups, respectively; *P* = 0.056). The serum ALP and γGTP levels were also investigated to evaluate the efficacy of the biliary drainage. Although the ALP and γGTP level at Day 0 and Day 4 had no difference between the two groups, the ALP and γGTP decrease rates were significantly higher in the 7 Fr catheter group than in the 5 Fr catheter group (ALP; 34.3 ± 2.1% vs 20.3 ± 2.6% *P* < 0.001, γGTP; 41.8 ± 2.0% vs 23.2 ± 5.8% *P* = 0.004). The success rate of ALP decrease tended to be higher in the 7 Fr catheter group, although the difference between the two groups did not reach statistical significance (98% vs 86% for the 7 Fr and 5 Fr catheter groups, respectively; *P* = 0.059). All three parameters (TB, ALP, and γGTP) showed similar trend (Table 2). The volume of drained bile did not differ significantly between the two groups (338 ± 29 mL vs 396 ± 42 mL for the 5 Fr and 7 Fr catheter groups, respectively).

**Table 2:** **Jaundice relief by 5 Fr or 7 Fr nasobiliary catheter**

	5 Fr	7 Fr	*P*
Total bilirubin (mg/dl)			
Before biliary drainage (Day 0)	5.4 ± 0.5^#1^	5.8 ± 0.8	0.674
After biliary drainage			
Day 1	4.6 ± 0.6	4.2 ± 0.6	0.688
Day 4	3.4 ± 0.5	2.9 ± 0.5	0.502
Decreasing rate (%)^#2^	40.5 ± 4.2	53.0 ± 3.0	0.019
Success rate^#3^	44 (88)	49 (98)	0.056
Alkaline phosphatase (IU/l)			
Before biliary drainage (Day 0)	1262 ± 110	1356 ± 137	0.595
After biliary drainage (Day 4)	927 ± 71	859 ± 82	0.536
Decreasing rate (%)	20.3 ± 2.6	34.3 ± 2.1	<0.001
Success rate	43 (86)	49 (98)	0.059
Gamma-glutamyl transpeptidase (IU/l)			
Before biliary drainage (Day 0)	576 ± 51	723 ± 74	0.107
After biliary drainage (Day 4)	387 ± 29	410 ± 46	0.677
Decreasing rate (%)	23.2 ± 5.8	41.8 ± 2.0	0.004
Success rate	46 (92)	50 (100)	0.117
Amount of bile drainage (ml/day)	338 ± 29.9	396 ± 42.0	0.267

^#1^Mean ± standard error, ^#2^Decreasing rate: (Day 0 - Day 4)/Day 0 × 100, ^#3^Success rate: improved patients/all patients (%).

In patients with acute cholangitis (13 patients in the 5 Fr catheter group and 9 patients in the 7 Fr catheter group), the period to alleviation of fever was also investigated. The period in the 7 Fr catheter group tend to be shorter than in the 5 Fr catheter group, but it was not significantly different (Additional file 3: Figure S3).

From the time of the ENBD placement to the catheter removal, all patients were closely monitored for complications associated with the nasobiliary catheter (Table 3). In the 7 Fr catheter group, one patient had post-ERCP pancreatitis and another had papillary bleeding. The pancreatitis was graded as moderate in severity and the patient could not resume eating for 6 days. The papillary bleeding was attributed to endoscopic papillary balloon dilatation, which was performed during the procedure. It was graded as mild because blood transfusion was not required. Self-removal of the catheter occurred in one patient in each group, and spontaneous dislodgement was observed in one patient in the 5 Fr catheter group. The two patients who pulled out their catheter were over 80 years old and both incidents occurred in the middle of the night. The catheter kinked on day 4 after the procedure in one patient in the 5 Fr catheter group; this required removal of the catheter.

**Table 3:** **Complications of the placement of nasobiliary catheter**

	5 Fr (n = 50)	7 Fr (n = 50)	*P*
Acute pancreatitis	0 (0)	1 (2)	0.500
Intestinal bleeding	0 (0)	1 (2)	0.500
Self-removal of the drain	1 (2)	1 (2)	0.753
Spontaneous dislodgement	1 (2)	0 (0)	0.500
Kinking	1 (2)	0 (0)	0.500

Data were shown as the number of patients with complications (percentage).

A questionnaire survey was performed after removal of the catheter (Table 4). One patient in the 5 Fr catheter group and two patients in the 7 Fr catheter group were unable to answer the questions because of Alzheimer disease. According to the survey results, total discomfort was significantly greater in the 7 Fr catheter group than in the 5 Fr catheter group (3.9 ± 0.2 vs 3.2 ± 0.2, respectively; *P* = 0.018). The larger-diameter catheter also tended to be associated with more difficulty in eating (2.7 ± 0.2 vs 2.2 ± 0.1, respectively; *P* = 0.079). The following symptoms were observed: nasal haemorrhage, 1 patient (1%); sore throat, 15 patients (15%); nausea, 1 patient (1%); and caught on a nearby object, 2 patients (2%). However, the incidence of these symptoms did not differ significantly between the two groups. The survey also revealed other minor adverse events including rhinorrhoea, hoarseness, difficulty in face washing, difficulty in taking medicine, constant worry about the catheter, and fatigue. Because all of these adverse events were graded as mild, treatment with the nasobiliary catheter did not require interruption in any patient.

**Table 4:** **Questionnaire survey in 5 Fr and 7 Fr nasobiliary catheter groups**

	5 Fr Catheters (n = 49)	7 Fr Catheters (n = 48)	*P*
Total discomfort^#1^	3.2 ± 0.2	3.9 ± 0.2	0.018
Difficulty with eating^#1^	2.2 ± 0.1	2.7 ± 0.2	0.079
Nasal hemorrhage^#2^	1 (2)	0 (0)	0.320
Sore throat^#2^	6 (12)	9 (18)	0.376
Nausea^#2^	0 (0)	1 (2)	0.310
Caught on a nearby object^#2^	1 (2)	1 (2)	0.988
Other adverse events^#2^	10 (20)	10 (20)	0.481

^#1^Data are shown as mean ± standard error of visual analog scale scores (0, none; 10, maximum); ^#2^Data are shown as the number of patients with the adverse event (percentage).

## Discussion

Several studies have compared the effectiveness of biliary stents with various gauges for the treatment of obstructive jaundice [8, 11]. These studies have failed to demonstrate significant differences in drainage effectiveness between different stent gauges. For example, Kadakia et al. reported a tendency toward a greater decline in total bilirubin levels using larger-diameter stents, but the difference between larger and smaller stents was not statistically significant [12]. No previous study has evaluated the effectiveness of different gauge nasobiliary catheters for the relief of obstructive jaundice. A crucial difference between biliary stents and nasobiliary catheters is their length. Because nasobiliary catheters are much longer than biliary stents, problems that impede bile flow, such as tube kinking, are more likely to occur with nasobiliary catheters.

Our results demonstrated that endoscopic biliary drainage using a 7 Fr nasobiliary catheter relieved obstructive jaundice faster and more reliably than drainage using a 5 Fr nasobiliary catheter, and that the decrease rates of all three parameters, total bilirubin, alkaline phosphatase, and gamma-glutamyl transpeptidase, were higher in the 7 Fr catheter group than in the 5 Fr catheter group. Because 5 Fr catheters relieved jaundice relatively slowly, the success rate for relieving jaundice on day 4 in the 5 Fr catheter group was less than 90% in the TB and ALP level. The success rate of relieving jaundice tended to be higher in the 7 Fr catheter group than in the 5 Fr catheter group, although the difference between the groups was not statistically significant.

In clinical practice, we sometimes encounter kinking of small-diameter nasobiliary tubes but seldom in large-diameter ones. Kinked catheters occasionally require removal because of blocked bile outflow. In the present study, only one patient had a catheter kinking; this patient belonged to the 5 Fr catheter group. Although the incidence of kinking in this study did not differ significantly between the groups, the issue of kinking is important, and further studies with larger sample sizes may be necessary to elucidate this issue further.

Our results demonstrated no difference in procedure-related complications between the two sizes of catheter. By contrast, Ishigaki et al. reported that 4 Fr nasobiliary catheters caused less post-ERCP pancreatitis than 6 Fr nasobiliary catheters [9]. However, the total incidence of this complication in their study was 9.7%, which was much higher than the 1.0% we observed in the present study. The lower rate in our study may have been due to the high rates of EPBD/EST (78%) and prophylactic pancreatic stent placement (57%) in our patients and/or our inclusion of patients who had undergone ERCP in the past. Concurrent EPBD/EST or pancreatic stent placement may prevent post-ERCP pancreatitis in patients with nasobiliary catheters [13].

Although 7 Fr catheters caused greater total discomfort than 5 Fr catheters in our study, the occurrence of other adverse events did not differ significantly between the two groups. All of these adverse events were minor and did not lead to discontinuation of the nasobiliary catheter treatment prior to the pre-scheduled removal time. The level of subjective total discomfort for both sizes of catheter was lower than we anticipated prior to the study, although there was substantial variation between individuals. Future studies to determine how to predict in advance who is most likely to tolerate a nasobiliary catheter may be useful to help further reduce the discomfort associated with these catheters.

## Conclusion

**Figure 2:**
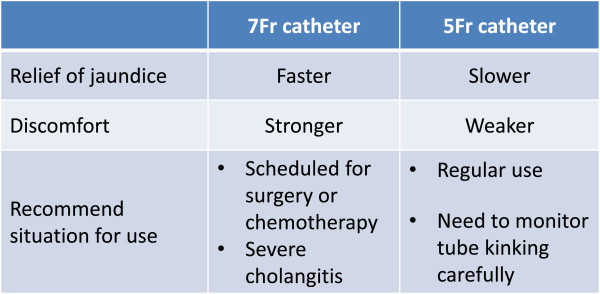
**Summary of the conclusion.**

The results of the present study revealed that 7 Fr nasobiliary catheters relieved obstructive jaundice faster and more reliably, but caused greater total discomfort than 5 Fr nasobiliary catheters. Therefore, 7 Fr nasobiliary catheters, despite their relatively minor disadvantages, are recommended for patients who require prompt relief of jaundice, such as those scheduled for surgery or chemotherapy or those with severe cholangitis. Conversely, smaller-diameter nasobiliary catheters are recommended if rapid relief of jaundice is not required, although tube kinking should be monitored carefully (Figure 2).

## Electronic supplementary material

Additional file 1: Figure S1: A questionnaire for ENBD catheter. (DOCX 28 KB)

Additional file 2: Figure S2: The bilirubin decrease rate in each primary disease. The bilirubin decrease rate in the 5 Fr or 7 Fr catheter groups was evaluated separately for each primary disease. The bilirubin decrease rate was higher in the 7 Fr catheter group in every primary disease. The blue and red lines represent 5 Fr and 7 Fr catheters, respectively. Data are expressed as mean ± standard error. * *P* < 0.05 compared to 5 Fr catheter group. (JPEG 87 KB)

Additional file 3: Figure S3: Time to alleviation of fever. In patients with acute cholangitis, the period to alleviation of fever after nasobiliary catheter placement was investigated in both groups. The result is shown in the Kaplan-Meier method and the difference is analysed by the Log-rank test. (JPEG 255 KB)

## Abbreviations

ENBD: Endoscopic nasobiliary drainage
TB: Total bilirubin
ALP: Alkaline phosphatase
γGTP: Gamma-glutamyl transpeptidase
Fr: French
EPBD: Endoscopic papillary balloon dilation
EST: Endoscopic sphincterotomy
VAS: Visual analogue scale.
