# Supplementary material for: Endoscopic nasobiliary drainage for obstructive jaundice using either a 5 Fr or 7 Fr catheter: a prospective, randomized trial
Source: BMC Gastroenterol. 2014 Sep 18;14:161. doi: 10.1186/1471-230X-14-161 (PMC4175279; doi:10.1186/1471-230X-14-161)
Supplement: Supplementary file 1 — Additional file 1: Figure S1: A questionnaire for ENBD catheter. (DOCX 28 KB) [file 12876_2014_1183_MOESM1_ESM.docx]

**Questionnaire on the nasobiliary catheter**

1. How uncomfortable did you feel with the nasobiliary catheter?

No discomfort Unendurable

　0 10

1. With the nasobiliary catheter, how difficult did you feel in eating?

No difficulty 　　 Impossible to eat

　0 10

1. Did you experience the following troubles?

・Nasal hemorrhage （　YES　・　NO　）

・Sore throat　 （　YES　・　NO　）

・Nausea, Vomiting （　YES　・　NO　）

・Caught by something around （　YES　・　NO　）

1. Please tell us about other troubles with the nasobiliary catheter, if any

Thank you for your cooperation

NTT Medical Center Tokyo

Department of Gastroenterology.
