# Supplementary figures and images for: Endoscopic nasobiliary drainage for obstructive jaundice using either a 5 Fr or 7 Fr catheter: a prospective, randomized trial
Source: BMC Gastroenterol. 2014 Sep 18;14:161. doi: 10.1186/1471-230X-14-161 (PMC4175279; doi:10.1186/1471-230X-14-161)

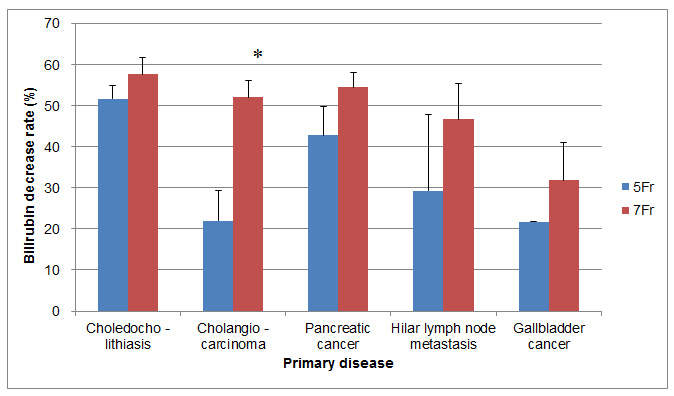

Supplement: Supplementary file 2 — Additional file 2: Figure S2: The bilirubin decrease rate in each primary disease. The bilirubin decrease rate in the 5 Fr or 7 Fr catheter groups was evaluated separately for each primary disease. The bilirubin decrease rate was higher in the 7 Fr catheter group in every primary disease. The blue and red lines represent 5 Fr and 7 Fr catheters, respectively. Data are expressed as mean ± standard error. * P < 0.05 compared to 5 Fr catheter group. (JPEG 87 KB) [file 12876_2014_1183_MOESM2_ESM.jpeg]

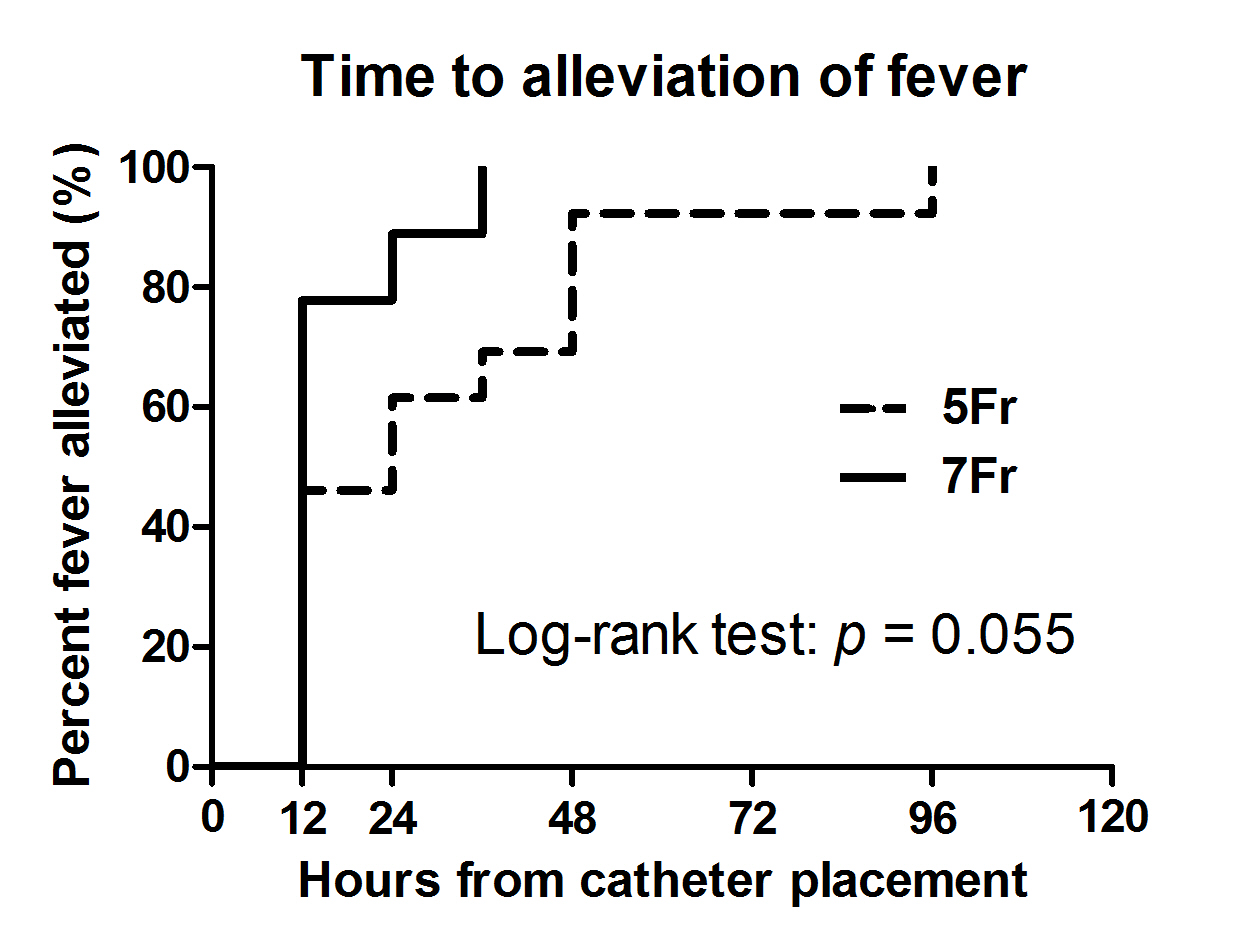

Supplement: Supplementary file 3 — Additional file 3: Figure S3: Time to alleviation of fever. In patients with acute cholangitis, the period to alleviation of fever after nasobiliary catheter placement was investigated in both groups. The result is shown in the Kaplan-Meier method and the difference is analysed by the Log-rank test. (JPEG 255 KB) [file 12876_2014_1183_MOESM3_ESM.jpeg]
